# Finding meaning in the Maze (Out): a co-produced reflexive thematic analysis of patients’ reflection tasks

**DOI:** 10.1186/s40337-026-01577-y

**Published:** 2026-04-03

**Authors:** Helene Nygaard Nielsen, Maria Mercedes Guala, Anette Søgaard Nielsen

**Affiliations:** Research Unit for Psychiatry Odense, Odense University Hospital, Odense, Denmark; University of Southern Denmark, Odense, Denmark, University of Southern Denmark, Hospitalsringen 90, 1st floor, 5260 Odense S, Denmark

**Keywords:** Eating disorders, Co-researchers, Patient involvement, Reflexive thematic analysis, Digital interventions, Maze Out, Self-reflection, Serious games, User perspective

## Abstract

**Background:**

Eating disorders (EDs) are complex psychiatric conditions often marked by difficulties in emotional awareness and self-reflection. Serious games (SGs) are emerging as digital tools that may support patient engagement and insight. Maze Out is a co-produced SG developed for EDs treatment, incorporating in-game reflection tasks. This study explores how patients with EDs responded to the reflection tasks in the SG Maze Out. Through this examination, we aim to illuminate how patients make sense of themselves and to generate insights that may inform the further development of therapeutic approaches.

**Methods:**

This descriptive qualitative sub-study of a randomized controlled trial analyzed 584 open-text responses from up to 133 patients with EDs from the Region of Southern Denmark, aged 18 years and older. Data was collected during gameplay and analyzed using reflexive thematic analysis. Five co-researchers with lived experience of EDs participated in two co-analytic workshops during the analysis to strengthen the interpretation.

**Results:**

Three overarching themes were identified: *A Mirror for Change*, *Ways Through*, and *Meeting Others*. Across these themes, patients reflected on decision-making, emotional regulation, and self-perception. Though often brief, responses indicated high emotional complexity and insight. Some described adaptive coping strategies and hopeful future perspectives, while others struggled with unmet needs and harsh self-evaluation.

**Conclusions:**

The findings suggest that the reflection tasks in Maze Out supported emotional processing, self-awareness, and hope among patients with EDs. The involvement of co-researchers contributed to a richer and more contextually grounded interpretation of the data. Maze Out may offer a meaningful supplement to traditional treatment by creating accessible and engaging opportunities for structured reflection.

**Supplementary Information:**

The online version contains supplementary material available at 10.1186/s40337-026-01577-y.

## Abstract (plain language summary)

This study explored how patients with eating disorders responded to written reflection tasks in the serious game Maze Out. Maze Out is a digital serious game developed with patients with lived experience of eating disorders and is designed to support treatment. The game invites patients to reflect on feelings, relationships, and everyday challenges. This study analyzed 584 reflection answers from patients who had played Maze Out during a larger research project.

Even short reflections gave essential insights. Patients reflected on personal needs, boundaries, social situations, and difficult emotions. Some wrote about using their eating disorder to manage feelings, while others described helpful strategies or hopes for a better future. For some, the game’s tasks pointed to real-life choices and dilemmas.

To better understand the reflections, the research team included five co-researchers who had experience with eating disorders. Their insights helped understand the reflections more deeply and ensured the results were relevant.

This study shows that reflection tasks in digital tools like Maze Out support emotional insight and self-understanding. While games like this are not a replacement for treatment, they can be a helpful extra tool. It also shows the value of involving people with lived experience in research.

**Supplementary Information:**

The online version contains supplementary material available at 10.1186/s40337-026-01577-y.

## Background

Eating disorders (EDs) are serious psychiatric disorders associated with substantial physical, psychological, and social impairment [1, 2]. Lifetime prevalence estimates indicates that anorexia nervosa (AN), bulimia nervosa (BN), and binge eating disorder (BED) affect approximately 8.4% of women and 2.2% of men worldwide [3, 4]. AN carries a particularly high mortality risk, with inpatients facing a fivefold increased risk of death compared to the general population [2, 5]. Timely and effective treatment is a public health priority. However, treatment engagement is often challenged by limited illness insight, ambivalence toward recovery, high dropout rates, and a fragile therapeutic alliance [6–11]. Additionally, patients frequently struggle with self-reflection and emotional awareness, making it difficult to articulate their needs or accept treatment [6, 7]. This emphasizes the need for supplementary treatment approaches that support emotional engagement and motivation [8]. One emerging strategy is to incorporate digital tools that promote self-reflection as an adjunct to treatment as usual [12, 13]. Self-reflection may stimulate consideration of one’s life situation and future perspectives, highlighting the potential value of interventions that support emotional engagement and self-understanding through accessible formats.

### Serious games

Serious games (SGs) are digital tools with therapeutic or educational aims, offering playful and immersive experiences designed to promote insight, learning, or behavioral change [14]. Their use in mental health contexts has expanded, particularly in relation to accessibility, stigma reduction, and the provision of flexible and discreet support [3, 15]. SGs have been suggested to offer emotionally safe environments for practicing new behaviors or reflecting on challenges, primarily based on findings from related mental health fields [16]. Within ED treatment, digital interventions have been discussed as potentially empowering tools that may support engagement and self-reflection, although evidence specific to serious games in ED populations remains limited [3, 17, 18].

### Self-reflection in eating disorder recovery

Self-reflection, the deliberate examination of one’s thoughts, feelings, and behaviours, has been described as an important psychological process, fostering insight and emotional regulation [19]. Enhancing self-reflection can initially evoke distress by bringing unprocessed emotions to awareness, but this process has been identified as a critical first step in recovery in mental illness [20]. In the context of ED, self-reflection becomes particularly relevant in addressing aspects of the illness that may be experienced as ego-syntonic, especially in conditions such as AN, where symptoms can be perceived as integral to the self, thereby limiting both insight and motivation for change [21–23]. In this light, self-reflection has been discussed as a potential pathway toward identity differentiation, emotional clarity, and behavioural change, central aims in ED recovery. In the context of Maze Out, self-reflection is therefore targeted as a core mechanism to support users in navigating these recovery processes.

### Maze Out

Maze Out is a digital SG developed at the Psychiatric Hospital in the Region of Southern Denmark in 2020 [24]. Together with software developers, it was co-produced by patients and clinicians, aiming to reflect lived experiences and promote user-centered design [11, 24]. Co-production has shown to enhance relevance and acceptability in mental health interventions [25–27]. Maze Out represents a user-informed approach integrating lived experience into its core structure.

When playing the game, patients navigate scenarios resembling everyday life, involving themes such as food, emotions, social interaction, and communication. The game includes reflection tasks, which prompt users to pause, consider their responses, and relate them to their daily lives [24].

Maze Out was evaluated among adult patients with ED in a randomized controlled trial (RCT), which examined its integration into treatment as usual (TAU) and assessed its impact [11]. The primary outcome of the RCT was changes in self-efficacy, with secondary outcomes including self-image, feelings of ineffectiveness and insecurity, and personal recovery after 15 weeks with access to the SG. The intention-to-treat and complete case analyses did not demonstrate a statistically significant effect on the primary outcome [11]. While prior work has addressed feasibility and acceptance, no study has yet analyzed how patients utilized the reflection tasks embedded in Maze Out or the nature of their responses.

The aim of the present study is to explore how patients with EDs respond to the reflection tasks in the SG Maze Out, using Reflexive Thematic Analysis to examine the meanings constructed in their responses.

Through this examination, we aim to illuminate how patients make sense of themselves and to generate insights that may inform and enhance therapeutic approaches.

## Methods

### Study design

This descriptive qualitative sub-study was embedded within the Maze Out RCT and focused on analyzing patients’ written responses to the game’s reflection tasks to better understand how adult patients engaged with and interpreted these components of the intervention. The analytic approach included structured co-analysis workshops together with co-researchers with lived experience of ED. The descriptive qualitative design supported an inductive and interpretive approach to understanding patients’ meaning making in response to the reflection tasks. The study is reported in accordance with the Consolidated Criteria for Reporting Qualitative Research (COREQ), a 32-item checklist designed to enhance transparency and rigor in qualitative health research [28].

### Setting and context

The original RCT included 133 patients, randomized to an intervention group who played Maze Out as an add to TAU up to 15 weeks or a control group, who was granted access to Maze Out after 15 weeks on a waiting list [11]. The participants in the RCT were adult patients with ICD-10 ED diagnoses receiving specialized treatment in outpatient mental health services across Denmark. Detailed information on eligibility, and study procedures is reported elsewhere [11, 29].

In Maze Out, patients encountered up to 38 distinct reflection tasks, depending on the chosen game path. The game was structured in missions. After each mission, the tasks were designed to prompt a therapist-led reflective exercise regarding the patient’s emotions and reactions that may have emerged during the mission, with the response registered in the game [11]. The reflection exercises were divided into 21 tasks that required open-ended text responses and 14 tasks with predefined response options, including optional free-text fields. Additionally, three reflection tasks allowed patients to opt out of missions related to overeating, compulsive exercise, and purging behaviors. This option was intentionally included to allow participants to pause or avoid content that might be experienced as emotionally challenging. An overview of the different types of reflection tasks is presented in Table 1.

**Table 1:** Reflection Task Types in Maze Out

Type of Reflection Tasks	Number of Tasks (*N* = 38)	Description
Open-ended reflection tasks	21	Require free-text input; users reflect on their emotions/reactions.
Tasks with predefined response options	14	Users choose from set responses; most include optional free-text fields.
Opt-out reflection tasks	3	Allow users to skip missions related to sensitive behaviors (e.g., purging).

### Data for analysis

In this study, the data consisted of patients’ responses to all 21 open-ended reflection tasks in Maze Out, totaling 584 text responses, from patients who had played the game during the RCT study. The present analysis focused exclusively on the 21 open-ended reflection tasks, as these generated self-produced textual data suitable for qualitative analysis of patients’ reflections. Tasks with predefined response options were not analyzed. Neither were the three reflection tasks aimed at allow participants to pause or avoid content included in the current analysis.

**Fig. 1:**
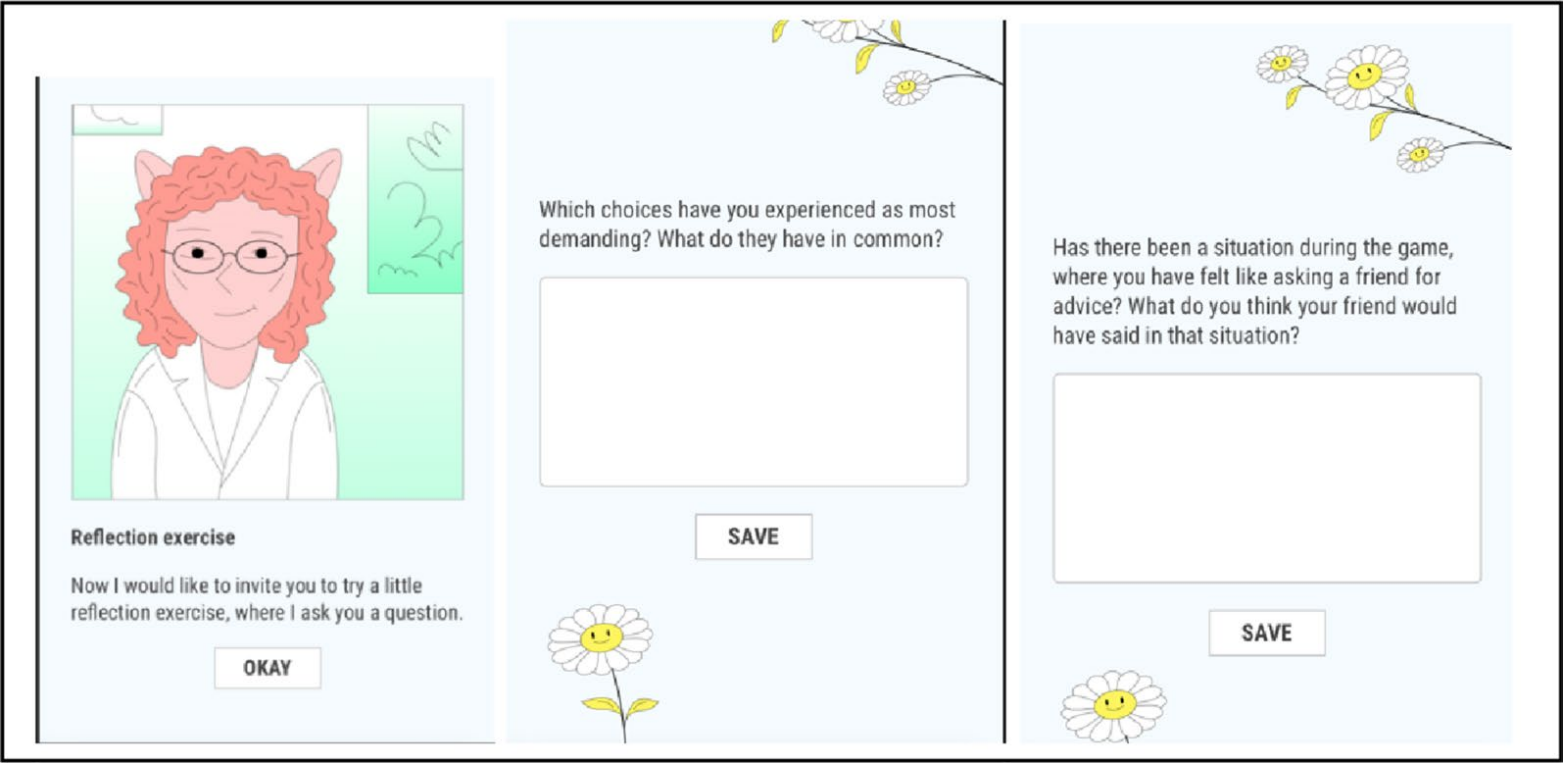
Example of reflection tasks

The reflection responses analyzed were drawn from both patients in the intervention group and the control group. The latter gained access to Maze Out after 15 weeks on a waiting list [11, 29]. All reflection task responses were extracted after completion of the RCT, in January 2025. Examples of the reflection tasks are presented in Fig. 1, and all reflection tasks are presented in Additional file 1.

The responses from the reflection tasks in Maze Out were collected pseudonymously [11]. Before the analysis, we anonymized all data by removing personally identifiable information and stored it in NVivo 1.7.1, which was used to manage and organize qualitative data [30]. Anonymization were conducted by the first author, HNN. The analysis was conducted in the original language, Danish. For the purpose of publication, quotations were subsequently translated into English. To preserve meaning across languages, translations were kept as close as possible to the original Danish wording, with attention to tone and nuance, and ambiguities were discussed within the research team when needed. The pseudonymous usernames could technically be linked to unique participant identifiers from the RCT; however, all usernames were removed prior to analysis. This implies that neither the research group nor the co-researchers had access to data identifying which individuals completed the reflection tasks. Consequently, it was not possible to determine whether individual patients contributed multiple responses to the same reflection task. Accordingly, the quotations in the analysis are not accompanied by references, as all reflection responses have been anonymized. 

### Data analysis procedures

#### Analytic approach and epistemological positioning

The data were analyzed using reflexive thematic analysis (RTA), which identifies, analyzes, and reports patterns within the material [31]. The RTA was conducted inductively, seeking to uncover latent themes that would illuminate underlying assumptions and ideologies. First author, HNN, epistemological position is grounded in social constructionism, viewing knowledge as situated and produced through social interaction [32]. To support this perspective, co-researchers with lived experience of EDs enriched the RTA by challenging HNNs preconceptions, with reflexivity acknowledged as integral to the analytic process. The co-researchers brought diverse lived experiences of EDs, including variation in age, treatment history (outpatient and inpatient care), stage of recovery, and illness duration. This diversity contributed to analytic reflexivity by enabling multiple interpretive standpoints during the co-analysis workshops. Differences in experiential positioning supported critical dialogue, challenged taken-for-granted assumptions, and enriched the interpretation of the reflection task responses, particularly regarding themes of ambivalence, hope, and self-understanding.

#### Patient involvement and co-analysis

Patient involvement was integral to the analytical process of refining and informing the RTA. Patient involvement as co-researchers followed the Involvement Matrix framework [33] and is reported using the GRIPP2-Long Form checklist [34].

A total of five female co-researchers with lived experience of EDs participated in the study. Eligible co-researchers were adults with lived experience of an ED who were either in the final phase of treatment or had completed treatment, were somatically stable, and expressed interest in contributing to qualitative research. Two were recruited through the Local Psychiatry Department in Odense, where clinicians introduced the study to eligible patients who were somatically stable and interested in research participation. The remaining three co-researchers were former research participants whom we had previously interacted with in various professional contexts. At the time of the study, three co-researchers were nearing the end of treatment, and two had completed it. Diversity in diagnosis and age was prioritized, and no one was excluded. The five co-researchers were aged 22, 23, 27, 41, and 57.

Two in-person workshops were conducted at the Psychiatry Department in Odense, with three and two co-researchers attending, respectively. Each lasted two to three hours and focused on identifying and discussing themes from the reflection task responses. Each workshop began with a comprehensive explanation and introduction to Maze Out, including its reflection tasks, followed by a review of the responses and initial theme development. Both workshops were facilitated and attended solely by HNN. The role of the co-researchers and the importance of their engagement was emphasized at both the beginning and conclusion of the workshops, as visualized and inspired by the Involvement Matrix. The Involvement Matrix is a framework for structuring roles and levels of influence in patient involvement [33]. They were positioned at the partner level according to the Involvement Matrix [33], indicating shared influence during the co-analytic phases of the RTA. While HNN conducted the initial familiarization and coding phases, the co-researchers played a decisive role in challenging, refining, and restructuring codes and themes during the co-analysis workshops. Differences in interpretation were addressed through collective discussion during the workshops, where divergent viewpoints were explored as analytic resources rather than resolved through voting or hierarchy, with HNN facilitating the dialogue and ensuring that alternative interpretations were documented and considered in subsequent analytic phases.

The co-researchers received a gift card valued at 200 DKK (approximately 26 USD) as compensation.

Analysis was carried out directly in NVivo 1.7.1, accompanied by HNN’s note-taking. The following section outlines how their knowledge contributed to the analytical process.

### Reflexive thematic analysis phases

Data were analyzed in Danish using Braun and Clarke’s RTA, following the six phases as documented in Additional file 2. An inductive coding approach was applied, incorporating both semantic and latent levels of meaning [31].

#### Phase 1–2: familiarizing and coding

HNN reviewed the data multiple times to gain familiarity, noting initial impressions during re-reading. HNN then inductively coded the material in NVivo 1.7.1, constructing patterns inductively without predefined categories. In total, 89 codes were identified and visualized in NVivo 1.7.1.

#### Phase 3: generating themes, co-analysis workshop one

In Workshop One the co-researchers were presented with the codes and their visualizations. HNN explained each code in detail, including suggestions for possible groupings. The co-researchers engaged critically, reflecting on how the codes resonated with their own experiences, sometimes supporting and other times challenging HNN’s proposals. This dialogue led to substantial rearrangements of codes and revisions of initial subthemes.

**Fig. 2:**
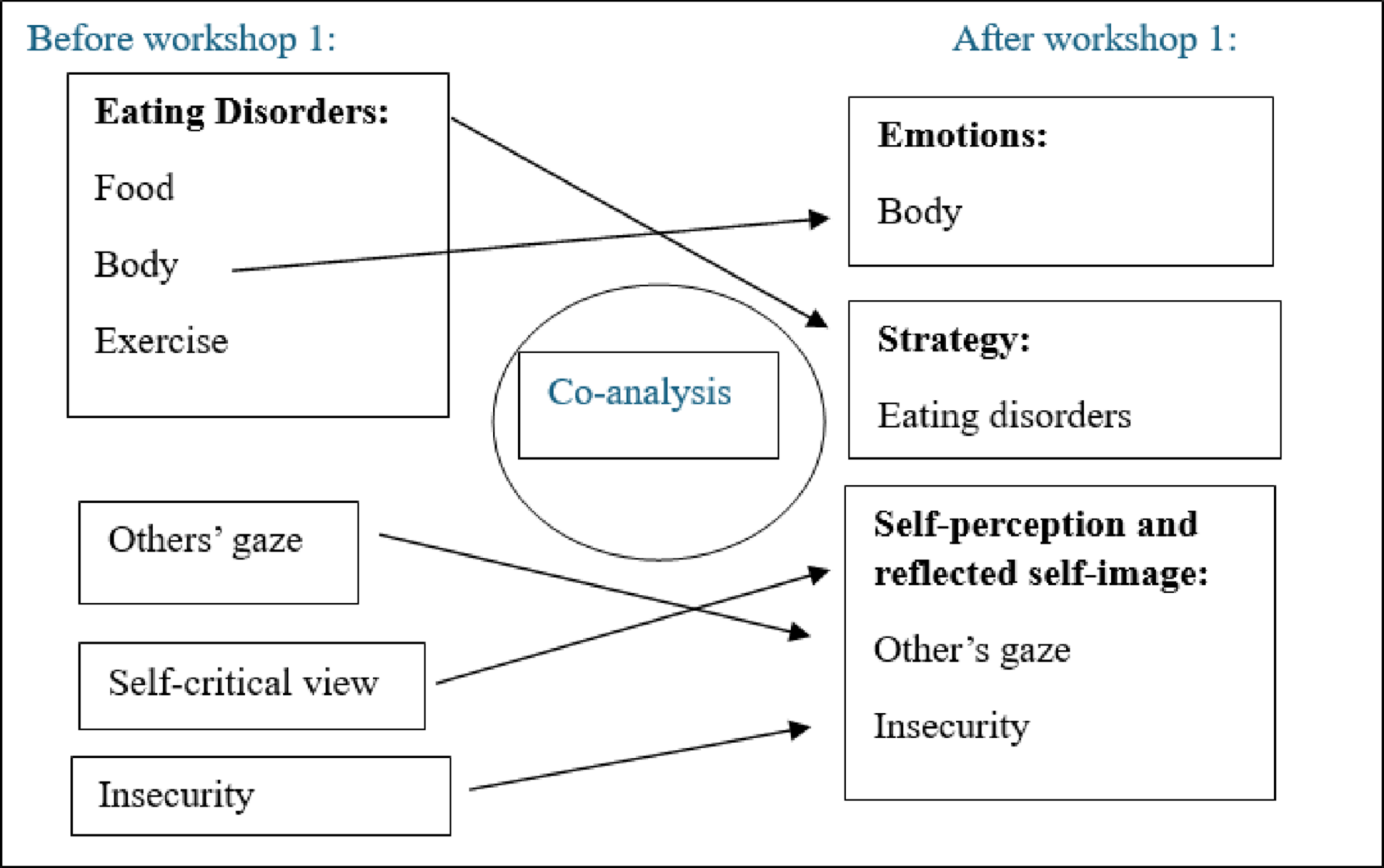
Example of co-analysis at Workshop One

For example, the co-researchers objected to placing the subtheme *“body”* under *“eating disorder”*. Instead, they argued it belonged under *“emotions”*, while *“eating disorder”* was reframed as a subtheme of *“strategy” which was situated within the overarching theme of "control"*. Similarly, the codes *“others’ gaze”*, *“self-critical view”*, and *“insecurity”* were consolidated into one theme. Co-researchers emphasized that *“others’ gaze”* reflects internalized self-perceptions rather than external opinions, highlighting how the ED shapes a critical self-image and unattainable ideals. They also pointed out that the ED was experienced as existentially necessary, making the prospect of recovery seem almost inconceivable (Fig. 2). By the end of Workshop One, six initial themes with corresponding subthemes had been identified; Ambivalence, Self-perception and reflected self-image, Future, Emotions, Control and Relationships (Additional File 2).

#### Phase 4: reviewing themes, defining and naming themes, co-analysis workshop two

**Fig. 3:**
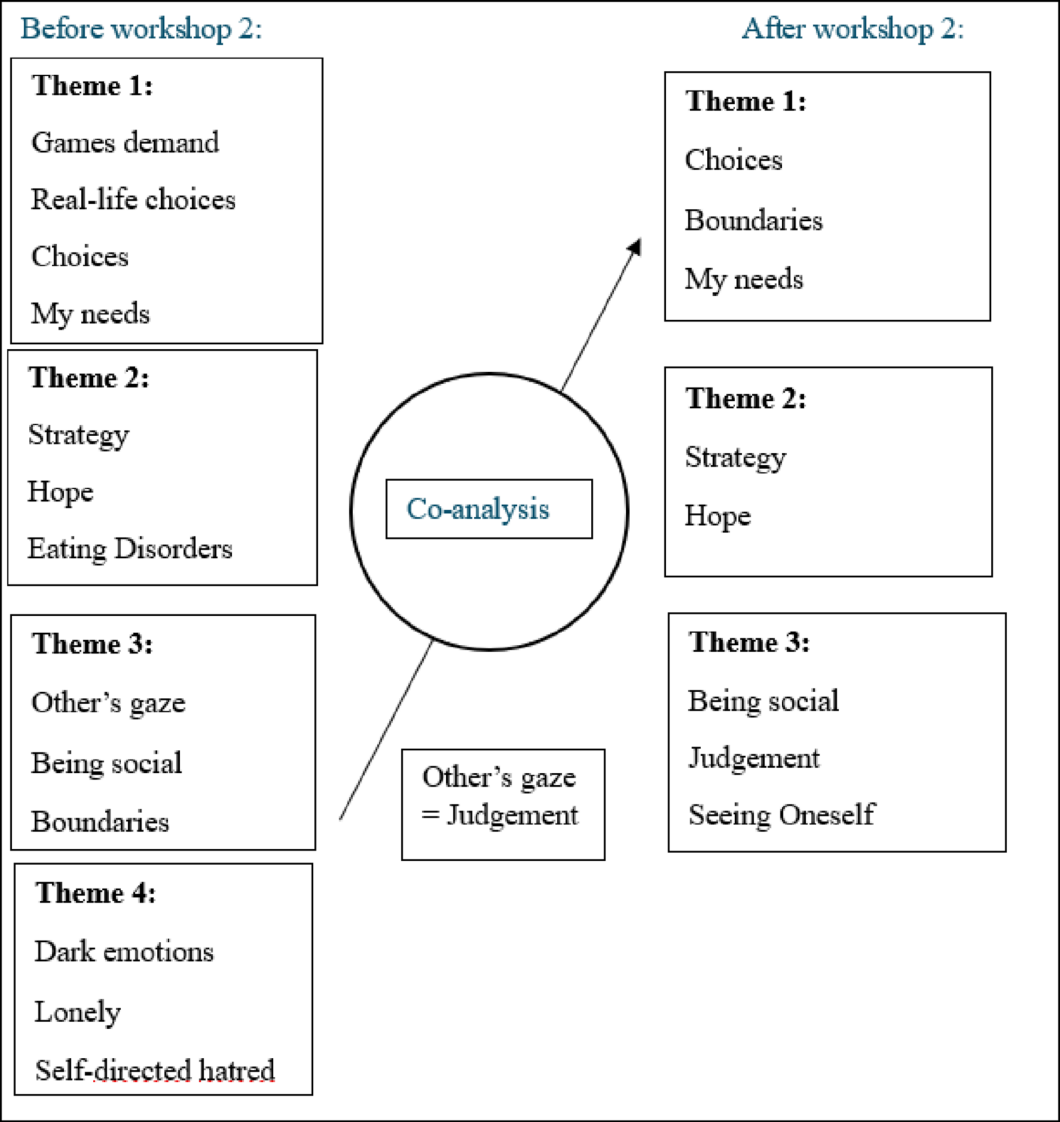
Example of co-analysis at Workshop Two

HNN then revisited all data, codes and themes, documenting suggestions for refinement. These suggestions were discussed and critically reviewed in the second co-analysis workshop. Together, HNN and the co-researchers critically reviewed the thematic structure, leading to several revisions. For instance, *“boundaries”* was reassigned from the social context to a theme on *“choices and needs”*. The number of overarching themes was reduced from four to three, *“dark emotions”* was removed as a standalone theme, and *“hope”* was elevated as a key subtheme, emphasizing the role of relationships in recovery. Workshop Two thus finalized Phase 4 (Fig. 3).

#### Phase 5–6: defining, naming, and finalizing themes

**Fig. 4:**
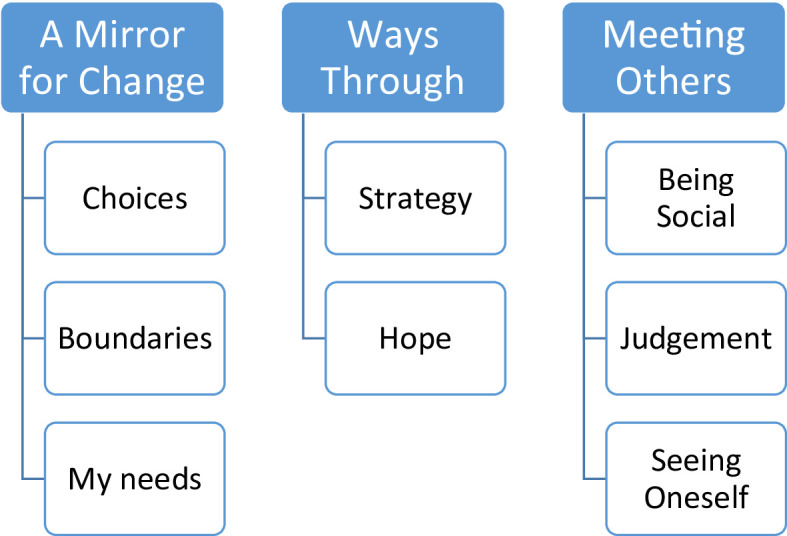
Themes and subthemes

Through the co-analytic process, three meta-themes with associated subthemes were established. While HNN conducted the final synthesis, definition, and naming of themes in Phase 5, this work was grounded in the prior co-analysis workshops, where co-researchers actively challenged, refined, and restructured codes and candidate themes. The final thematic structure was subsequently presented and discussed with the wider author group (MG, ASN), ensuring that multiple perspectives informed the analytic decisions. A flow diagram of the process can be seen in Table 2, and the finalized themes are presented in Fig. 4.

**Table 2:** Process of the RTA

Step	Activity	
1. Data Familiarzation	Read and re-read data, taking notes	HNN
2. Systematic Data Coding	Inductive coding of data, using NVivo 1.7.1.	HNN
3. Generating Initial Themes	Organized 89 codes, identified six main themes and subthemes	Co-analytic process at Workshop 1
4. Reviewing and Naming Themes	Reviewed and refined themes	HNN Co-analytic process at Workshop 2
5. Defining and Naming Themes	Defined and named themes, revised after discussions	HNN, MG, ASN
6. Writing the Report	Presentation of the results	HNN

### Strategies to ensure methodological rigor

To enhance the methodological rigor of the study, strategies addressing credibility, transferability, dependability, and confirmability were applied, in line with established criteria for qualitative research [35]. Credibility was supported through the use of RTA, iterative engagement with the data, and structured co-analysis workshops with co-researchers with lived experience of EDs, who actively challenged and refined codes and themes. Transferability was strengthened through detailed descriptions of the study context, the reflection tasks, and the analytic procedures, enabling readers to assess the relevance of the findings to other settings. Dependability was addressed through transparent documentation of analytic decisions across the phases of analysis, supported by the systematic data management and coding in NVivo 1.7.1, including screenshots illustrating coding, theme development, and refinement across the RTA phases (Additional file 2). Confirmability was enhanced through reflexive positioning of the first author, collaborative discussions with co-researchers and the author group, and ongoing attention to grounding interpretations in the empirical material rather than individual assumptions.

## Results

Most reflection responses were brief, often consisting of only a few words or a single sentence, and are presented in quotations. Although many reflection responses were brief, they provided sufficient analytic depth when examined across the dataset. As a result, the presentation of findings relies on concise quotations supported by an interpretive analytic narrative. The RTA identified three overarching themes: *A Mirror for Change*, *Ways Through*, and *Meeting Others*. Each theme and associated subthemes are presented below (Fig. 4).

### A mirror for change

The game’s reflection tasks invited patients to confront difficult choices and increase their awareness of personal boundaries and unmet needs. Patients described the reflection tasks as a space for insight that encouraged pausing, reflection, and consideration of parallels between in-game decisions and real-life challenges.

### Choices

In Maze Out, patients were continuously confronted with choices, which several patients found difficult. These choices prompted reflection on decision-making in their own lives, particularly regarding which decisions they found most challenging, thereby emphasizing the perceived complexity and emotional burden of making choices.

Choices about food.

It’s hard to make decisions because the eating disorder makes me doubt everything.

Choices involving other people.

What’s best for me, what will make me happiest.

The variation in responses to the tasks indicated that decision-making was challenging on multiple levels. This included seemingly simple, everyday choices, such as food decisions that might take others only moments to make. It also extended to more fundamental and existential choices, such as identifying what brings joy. These examples are presented as analytically representative of the broader variation in the material rather than as an exhaustive account of all types of decision-making challenges described.

Another task prompted patients to describe their experience of being repeatedly confronted with choices in the game.

I am forced to choose, and that’s a good exercise.

It’s hard to make decisions, but it’s also challenging and a bit exciting.

A good opportunity to reflect on my choices in real life.

I’m not quite sure. I wish I could act out the good decisions in real life, too.

Despite the fictional game, some patients playing Maze Out experienced making choices in the game as demanding. Others viewed it as a helpful practice, though they also recognized the challenge of transferring practice into real-life contexts. For some, the reflection inspired motivation to improve decision-making, while others highlighted their struggle. This challenge was particularly evident concerning social boundaries.

I am most challenged by the choices involving social interactions, setting boundaries….

Those involving food and social interaction.

### Boundaries

Patients frequently illustrated difficulties identifying and respecting their boundaries throughout their reflections, especially in social contexts. Parties, for instance, were described as situations in which personal boundaries blurred. These reflections were generated in response to a task asking patients to imagine attending a party and to describe challenges or obstacles they feared encountering.

When I drink, I find it really hard to feel my limits.

...when your boundaries are not respected.

Struggling to maintain boundaries was not limited to uncertain or new social contexts; patients reported giving in rather than asserting themselves even within close family relationships.

When you’re eating with family, and they want you to take more. It’s hard to say no, so you just give in.

Giving in was sometimes experienced as necessary in family contexts, particularly during meals, where different interests and dynamics around food may emerge. Patients also described boundary-setting difficulties in education environments in response to a reflection task asking how they experienced working together with others following a study-related mission.

*It can be uncomfortable to stand firm in group work…*

Challenging and demanding to set boundaries.

I don’t like saying no.

Across responses, setting boundaries appeared as a central struggle, whether with friends at parties, family members, or peers in formal settings like study groups. One patient suggested in her reflection that difficulty in boundary-setting stems from not being in touch with one’s needs.

Saying no when there’s something I don’t want or have the energy for. It’s nearly impossible for me, and I’m bad at listening to my own needs.

### My needs

Several patients expressed difficulty identifying and responding to their needs in their reflection tasks. These reflections were generated in response to a task asking patients how they feel about canceling plans, for example with friends.

I’m generally terrible at saying no and checking in with my needs.

Meeting my needs.

Some noted that their needs were complex to identify in the presence of others.

I always find it hard to choose something different than others. I think the same as them, and it really annoys me.

While some struggled to access their needs in social settings, others described positive experiences of prioritizing themselves, such as cancelling plans.

It feels good because I just don’t want to or have the energy to meet up, so I feel like I’m listening to myself. Others have to accept that.

I’m fine with it. It’s important to show care for yourself.

Responses revealed a wide variation in patients’ connection to their needs. Some could acknowledge and act on their needs about social commitments, while others struggled with identifying even their most basic needs. These reflections were generated in response to a task asking patients to identify which types of choices they found most challenging.

Eat or not eat? Exercise or not exercise? Work or rest?

Exercise or relaxation? Food or fasting?

Sleep.

The reflections varied in the degree to which patients could identify their needs. Some responses suggested that patients had struggled to navigate personal needs in social contexts and instead mirrored others due to a lack of internal awareness. Others indicated that they had been able to recognize and act on their needs, such as cancelling social engagements when necessary. For some, the primary challenge was identifying basic physical needs like rest, food, and sleep, which, when unmet, were profoundly distressing.

### Ways through

This theme captured how patients described their use of coping strategies, both helpful and harmful, when navigating emotional challenges. The reflections highlighted the significance of hope and personal goals, and how ED was often used as a means of emotional regulation without alternative strategies.

### Strategy

Some reflection tasks invited patients to consider which strategies to use to get through difficult situations or challenging days. Several patients responded that being with others, and in some cases talking about their difficulties, served as helpful coping mechanisms.

When I’m with them, the illness takes up less space.

…I try to talk about it with those close to me, even though it’s incredibly difficult.

Saying it out loud, trying to find the root of the feeling.

When short-term coping strategies were described, the responses varied significantly. Many patients mentioned self-injurious behavior or disordered eating patterns as unhealthy strategies.

…hiding under the blanket or self-harming. I increase my use of laxatives in the days that follow.

…I can get so frustrated that I hurt myself, often hitting myself ‘lightly’.

Distraction, extra laxatives, occasionally self-harming, eating less, isolating myself, contacting a friend.

For several patients, the ED itself was used as a primary coping mechanism in response to emotional distress. In this context, disordered behaviors were deemed necessary for emotion regulation. The absence of this strategy was experienced as a difficulty.

…not being allowed to use the eating disorder to regulate my emotions.

In contrast, some patients used more long-term strategies that reflect trust in their current treatment. Despite not yet feeling better, they expressed faith in future change.

Surrendering to and trusting that the eating disorder treatment I’m undergoing will eventually help, even though I don’t feel any change yet.

Both adaptive and maladaptive strategies were described in the reflection responses, underscoring how everyday life with an ED could be profoundly distressing when such varied strategies were employed.

### Hope

While the reflections on strategy sometimes implied a reliance on the ED, hope was constructed as a vital component in patients’ ability to envision recovery. Hope was described as necessary for enduring difficult times.

It will be okay, it will get better someday, hold on.

Tomorrow is another day.

Other responses revealed that some patients not only hoped for better days but also imagined an improved everyday life.

I dream of a life where I have a job. My colleagues like me, and we have coffee breaks and eat lunch together.

Such visions of ordinary routines, taken for granted by others, are remarkable. They suggest the patient’s current reality is shaped by a struggle that makes these simple aspirations deeply meaningful. Expressing hope for a future where the ED plays a less dominant role or might even become a thing of the past.

I hope to survive my anorexia.

…to learn to live with the eating disorder.

…that I no longer need psychiatry and can refer to my eating disorder as ‘when I had an eating disorder’.

To make choices based on desire and my needs instead of rules and calculations.

These responses reflected a broad spectrum of hope, from mere survival to adaptation to complete recovery. One patient articulated a more pragmatic hope: freedom from the disorder’s rigid rules and rituals. Others envisioned a future that included meaningful relationships and family life.

To make plans for my life with my husband.

To have my own family.

A better everyday life with my children.

To get my eating disorder under control so I can, among other things, be more social.

The desire to plan for the future, to imagine having a family, or to improve day-to-day life with one’s children summarizes how essential hope is. Such aspirations reflect the importance of social connections, which are often a critical component in healing from EDs.

### Meeting others

This theme covered patients’ social interaction experiences, self-perception, and judgment. Reflections revealed the emotional cost of engaging with others, the internalization of negative self-images, and the complex dynamics of navigating relationships while managing EDs.

### Being social

Spending time with others was described in the reflection tasks as both enjoyable and exhausting. Several patients reflected that social interactions could be pleasant but emotionally draining, often leaving them fatigued for days.

It’s nice to be with other people. But also nice to be able to withdraw when my energy is spent. Unfortunately, I’m exhausted for days afterward.

The mental effort required in social settings may partly be explained by patients’ heightened self-awareness and concern about how others perceive them.

Worried I said something wrong or was too much.

That I look stupid or fat.

I struggle to talk to people because I get nervous and insecure.

Responses suggest that being social in settings with more than a few trusted individuals is particularly challenging. Some described feeling lonely or out of place in such situations.

That I wasn’t good enough at creating a nice atmosphere.

Standing alone and looking lonely, having no one to talk to.

Feeling lonely among all the people.

Patients reported feeling uncertain or insecure even in structured environments such as educational group work.

I often feel insecure about my own abilities.

I often feel insecure when working with others, I think a lot about what they think about me and my input.

I prefer working alone so I don’t have to be judged by others.

The reflection responses indicated that several patients found it highly demanding to be in social contexts and felt responsible for the atmosphere. Feelings of insecurity and loneliness in social situations were mentioned in multiple responses, suggesting that patients often struggled to imagine what others expected of them in these interactions.

### Judgement

As indicated in many of the responses, the imagined expectations or thoughts of others may have pointed to a recurring fear of being judged or evaluated.

Being judged by others.

I feel exposed, judged, and evaluated by people.

This perceived judgment appeared to fuel a belief among some patients that others did not enjoy their company or thought poorly of them.

Afraid of… what others think of me.

…that people find me boring.

Although there were no clear indications that patients had directly experienced negative judgments, some noted that subtle cues, such as looks or glances, could be very telling. One patient even expressed concern that others could read their thoughts.

Questions and looks.

…I wondered if they could read my thoughts.

While patients worried about what others thought of them, these fears were arguably overshadowed by the harsh ways in which they viewed themselves.

### Seeing oneself

Patients answered questions about their self-perception in various contexts through reflection tasks. Their responses indicated that they often compared themselves to others.

Comparing myself with….

In one of the reflection tasks, patients were asked to describe something they liked about their body. Some responded that there was nothing they liked.

There’s nothing I like.

Only a few expressed positive feelings about their appearance, mainly related to specific features. However, no patient mentioned being content with their body’s functions.

I like my face and hair… I try to find something I like about my body.

I have a good relationship with my body.

I like my eyes and my breasts.

Most responses, however, reflected deep dissatisfaction with their bodies.

I see a big, saggy lump of fat.

I don’t see a person. Just a huge blob.

A big disgusting body that is wrong.

I hate the way I look.

These intensely negative self-evaluations provide a stark glimpse into the emotional and cognitive experiences associated with EDs. One patient’s statement offers a powerful summary of this internal turmoil.

A confused and insecure girl. A lack of joy and spark, I don’t recognize myself.

## Discussion

This study describes the reflections provided by adult patients with EDs while playing the SG Maze Out as an adjunct to TAU. Through an RTA, the analysis explored how patients responded to the reflection tasks in the game. Although many answers were brief, they conveyed significant emotional depth. This conciseness may indicate the limitations of the mobile game format for extended written expression or patients’ difficulties articulating needs [6, 7]. The analysis generated three overarching themes: *A Mirror for Change*,* Ways Through*, and *Meeting Others*, capturing how the game facilitated reflection on choices, boundaries, needs, coping, and self-perception. Notably, participants varied in how they experienced the reflection tasks; Some found them emotionally challenging, while others perceived them as meaningful prompts for insight and future-oriented reflection. This variation may reflect differences in patients’ readiness for change and emotional capacity, suggesting that individuals engaged with the reflection tasks from varying psychological starting points.

These findings align with results from a previous sub-study of Maze Out, in which patients were interviewed about their experiences of playing the game. That study supported the present interpretation by showing that the game’s playful and validating structure was perceived by patients as offering a non-judgmental space to explore their inner world [36]. Guala and colleagues [37] concluded that the game facilitates insight, hope, and the sense of being “good enough”, experiences also evident in the reflection responses in the present study. The co-produced design and mentalization-informed framework likely contributed to emotional processing and dialogue between patients and relatives [37]. These findings support the interpretation that even brief reflection responses can carry substantial meaning and thus therapeutic potential, which highlights the importance of including sparse expressions in the analysis of this study.

Compared to previous interventions, which have often targeted AN or broader mental health populations using video- or VR-based formats [7, 38, 39], Maze Out represents a co-produced, reflection-based SG specifically designed for a broader group of adult patients with EDs [29]. The game’s reflective tasks are intended to support processes related to emotional awareness, insight, and motivation for change, factors widely recognized as therapeutic targets in ED care [40, 41].

This qualitative analysis adds depth to the evaluation of SGs, particularly Maze Out, by illustrating how its reflection components may contribute to these outcomes. Structured self-reflection and personalization may be key in supporting engagement and emotional processing in digital interventions. Notably, personalization and usability have been identified by ED clinicians as some of the most important design principles for successful implementation [42].

The present study highlights the potential of SG, such as Maze Out, to engage patients with EDs in reflection-based processes that can foster self-awareness and support therapeutic goals. Patients’ reflections revealed that the game evoked emotional responses, heightened awareness of personal needs and boundaries, or the absence thereof, and opened space for reconsidering coping strategies.

These findings are consistent with existing literature that emphasizes the importance of personally relevant and engaging digital interventions in the treatment of EDs [43]. The present study demonstrates how the reflection tasks provided a space for patients to explore themes related to regulating their emotions, identity, and social relationships, which are often challenging for patients with EDs.

With its reflective tasks and co-produced game structure, which facilitates personalization, Maze Out may support a sense of relevance and relational connection, even without direct therapist contact. A recent Delphi study supports this perspective: Lemmer et al. [42] concluded that digital mental health interventions are most effective when they combine engaging strategies with user-involving design and a high degree of personalization. Furthermore, Vajawat et al. [44] emphasized the lack of therapeutic alliance in digital formats as a key barrier to patient motivation. However, such interventions should not stand alone. Tang et al. [7] argue that SGs can lower the barriers to treatment and be a more accessible form of treatment than conventional treatment, as there are no barriers such as trust in the therapist or other usual avoidance issues.

The reflection responses in Maze Out, particularly those related to strategies and hope within the theme *Ways Through*, illustrated how SGs can make challenges visible and point toward potential paths forward. In the present study, even simple reflection tasks activated hope and future-oriented thinking, such as imagining relationships, employment, or life without an ED. These findings also echo the conclusions of Thompson et al. [45], whose study found that digital interventions which promote visualization of positive changes and can help sustain motivation in the recovery process.

The reflection tasks in Maze Out were developed as a supplement to ED treatment. Borghouts et al. [46] emphasize that integration into daily life and sustained engagement are crucial to the effectiveness of the intervention. This suggests that SGs are best understood as supplements, rather than replacements for existing treatment options. This interpretation is supported by Hartmann et al. [47], who argue that digital interventions are most effective when combined with other forms of psychosocial support. Similarly, Lemmer et al. [42] emphasize hybrid models as the most promising approach for achieving outcomes.

A key feature of the present study was the involvement of individuals with lived experience of EDs as co-researchers in the RTA. This approach aligns with the growing focus on participatory methods in ED [45]. Thompson et al. [45] emphasize how lived experience contributes to a deeper understanding of recovery, identity, and social dynamics. In the present study, co-researchers’ input was vital for interpreting and categorizing themes such as personal needs and strategies. Their perspectives enriched the analysis and ensured that the final themes reflected the complexity of patients’ responses more accurately.

### Strengths and limitations

This study has limitations to consider when interpreting the findings. The reflection task responses were fully anonymized, so it was not possible to determine whether individual patients contributed multiple responses, limiting our ability to establish the number of unique participants represented in the dataset.

Furthermore, it is unclear which patients from the original RCT study submitted responses and what influenced their participation. Still, a strength is that the reflections were collected directly during gameplay, enhancing naturalistic context. The RTA was conducted with co-researchers with lived experience of EDs, who, as agreed from the outset, participated anonymously. While this constituted a limitation, as their subjectivity and background could not be presented, their participation nonetheless represented a methodological strength by enhancing analytic depth and ensuring contextual relevance. This study used the structured and transparent reporting of patient involvement in the GRIPP2-Long Form checklist [34]. This contributes to methodological clarity and helps articulate the significance of co-researchers’ contributions.

The involvement of co-researchers in the analysis of the patients’ reflection is considered the core strength of the present study. However, it needs to be acknowledged that although partner-level collaboration was supported during the co-analytic phases using the Involvement Matrix [33], power asymmetries between academic researchers and co-researchers cannot be fully eliminated and this should be considered when interpreting the findings. 

## Conclusion

Maze Out seems to facilitate self-reflection on emotional regulation, identity, and social relationships, domains often challenging in ED treatment. Even the brief written responses revealed meaningful insight and showed how short reflections can carry significant therapeutic value. The game may thus serve as a meaningful supplement to existing treatment approaches by supporting patient engagement, motivation, and insight. Involving co-researchers with lived experience enriched the analytic process and ensured that the themes reflected patients’ realities.

## Supplementary Information

Supplementary Material 1

Supplementary Material 2

## Data Availability

The datasets are not publicly available due to participant anonymity but are available from the corresponding author on reasonable request.
