# Supplementary material for: Finding meaning in the Maze (Out): a co-produced reflexive thematic analysis of patients’ reflection tasks
Source: J Eat Disord. 2026 Apr 3;14:113. doi: 10.1186/s40337-026-01577-y (PMC13173963; doi:10.1186/s40337-026-01577-y)
Supplement: Supplementary file 1 — Supplementary Material 1 [file 40337_2026_1577_MOESM1_ESM.docx]

# Additional file 1: Reflection Tasks in Maze Out (Translated from Danish)

1. Think about the last time you attended a party. What experience do you recall, and what emotions does it evoke?
2. Imagine you are going to a party. Name the challenges or obstacles you fear encountering.
3. How has it affected you to be confronted with demands during the game?
4. Was there a situation in which you felt like asking a friend for advice? What do you think your friend would have said in that situation?
5. Try to pause and close your eyes. Focus your attention on your body for one minute. Then open your eyes and continue. How does it feel, strange, pointless, pleasant, buzzing? Do you know where restlessness resides in your body when you are sad/nervous/afraid? Is the location the same, or perhaps all over?
6. Think of one good thing from your day today. What was it?
7. What do you like about your body? What do you dislike? What captures your attention?
8. What does your appearance mean to you?
9. If you feel unwell, cold, tired, struggling with memory and focus in daily life, what do you do?
10. When you look at yourself in the mirror, what do you see? And what do you think about what you see?
11. How do you feel about canceling plans, for example with your friends?
12. What strategies do you use when you don’t feel good enough or attractive enough?
13. Try to give yourself a compliment. It could be for something you’re good at, something about your body, or something you’ve done today. It can be a small thing.
14. Think about a time when you were feeling low. What would you say to a friend who felt that way?
15. What are your dreams for the future?
16. Now that you have played the game for a while, have your dreams for the future changed? If yes, what are they now?
17. How do you experience going shopping for clothes with a friend?
18. How do you experience working together with others?
19. Can you identify which types of choices are most challenging for you?
20. What real-life situation does this dream remind you of?
21. Which choices have you found most demanding? What do they have in common?
