# Supplementary material for: Finding meaning in the Maze (Out): a co-produced reflexive thematic analysis of patients’ reflection tasks
Source: J Eat Disord. 2026 Apr 3;14:113. doi: 10.1186/s40337-026-01577-y (PMC13173963; doi:10.1186/s40337-026-01577-y)

# Additional file 2: Overview of the RTA in NVivo 1.7.1

**Figure 1**: The first five phases of the RTA were conducted using NVivo 1.7.1.


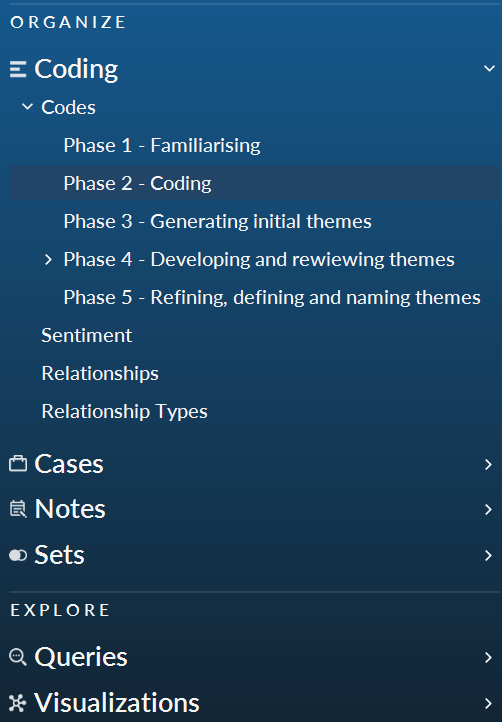


**Figure 2:** Overview of the coding process in Phase 2 of the RTA. The coding resulted in 89 distinct codes.


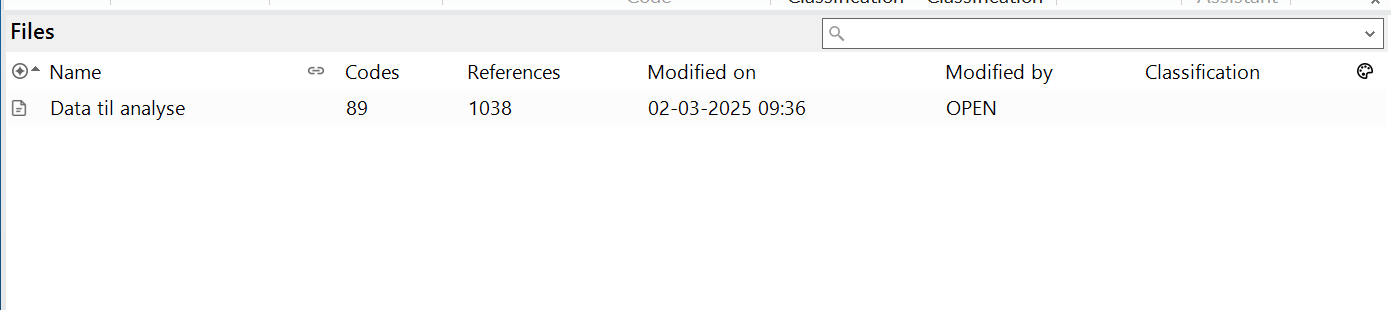


**Figure 3:** Mind map of themes and subthemes in Phase 4 prior to Workshop 2.


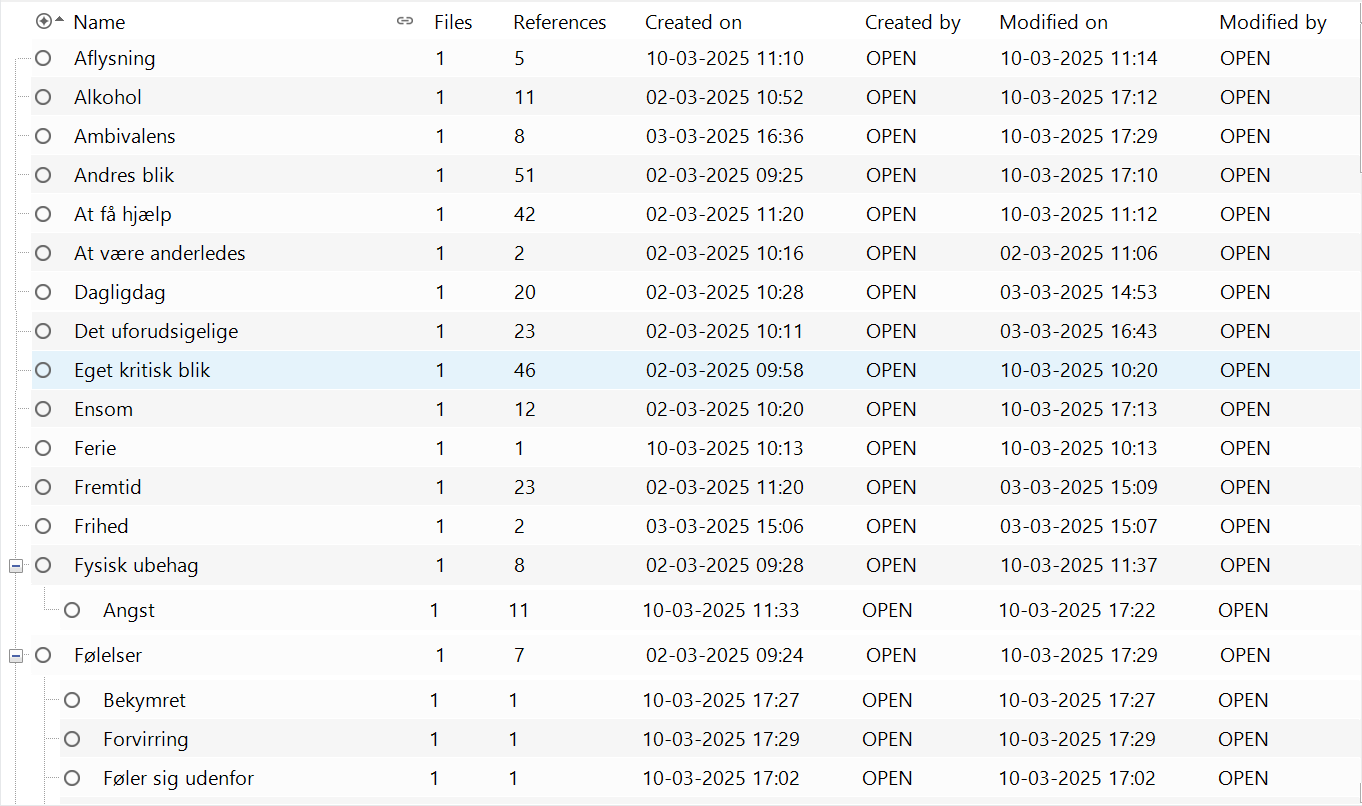


**Figure 4**: Illustration of the 89 codes prior to Workshop 1 and Phase Three of the RTA


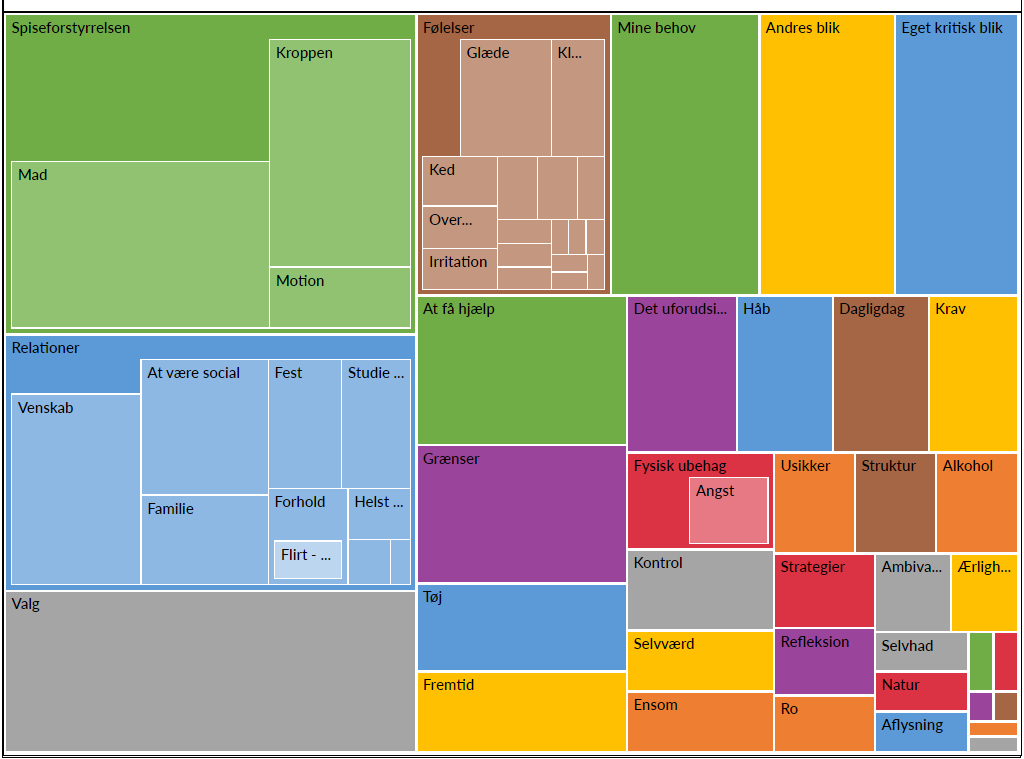


**Figure 5:** The six themes and subthemes following Workshop 1 and Phase Three of the RTA


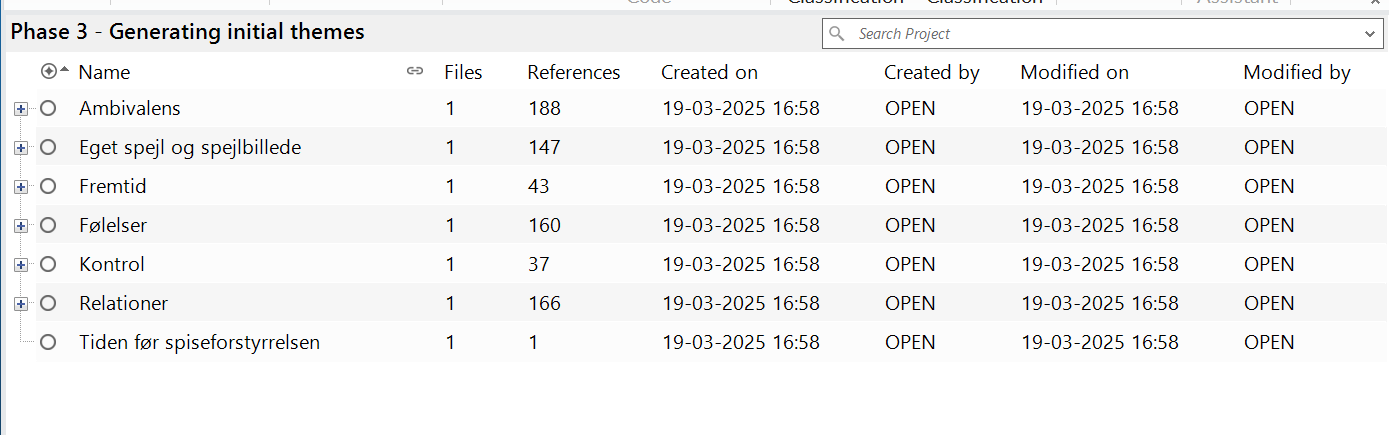


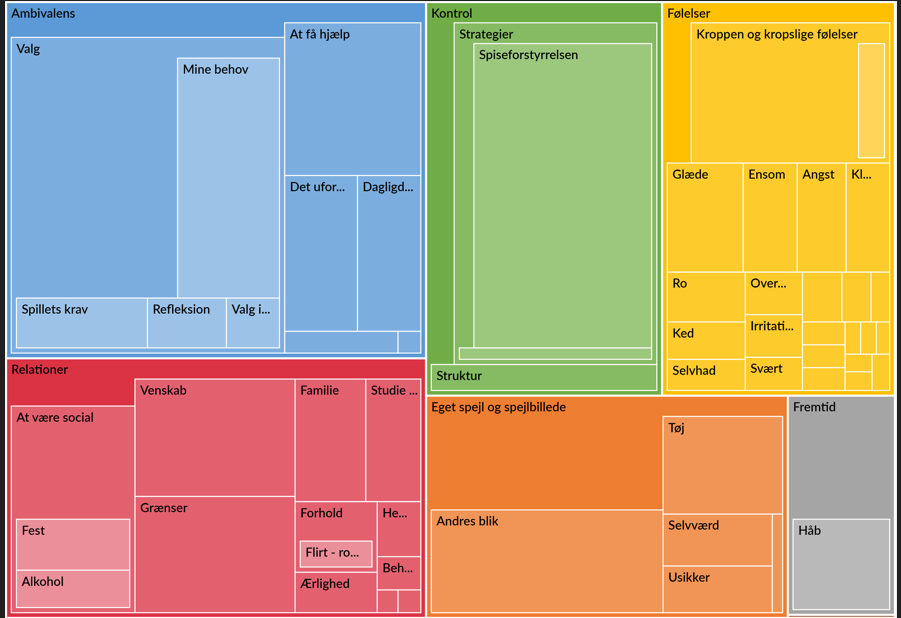


**Figure 6:** Mind map of themes and subthemes in Phase 4 prior to Workshop 2.


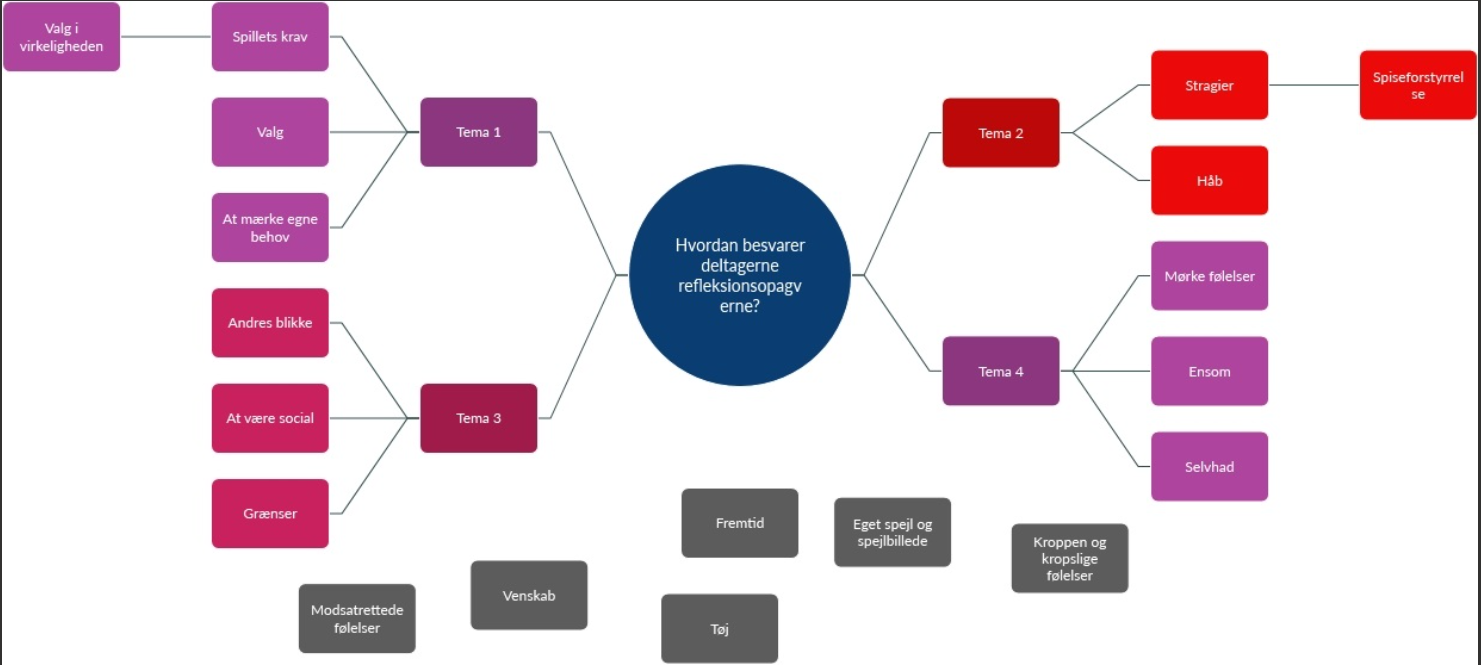


**Figure 7:** Mind map of themes and subthemes in Phase 4 after Workshop 2


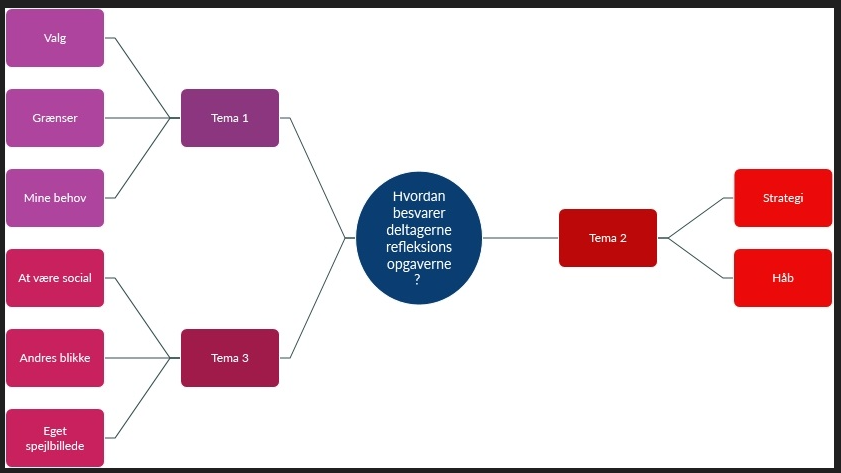


**Figure 8:** Themes and subthemes at phase 5


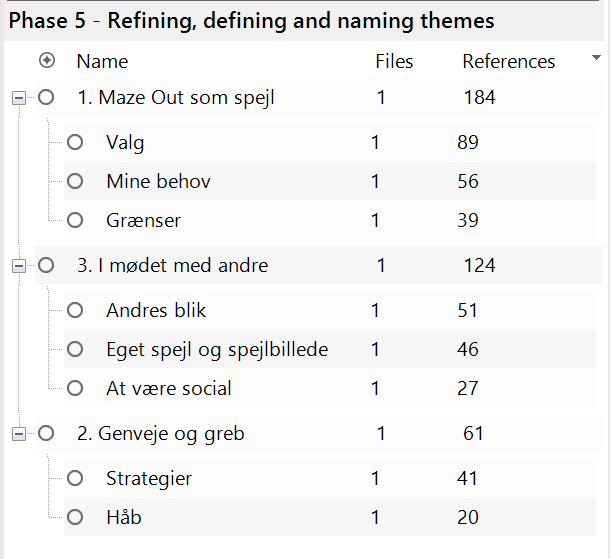

Supplement: Supplementary file 2 — Supplementary Material 2 [file 40337_2026_1577_MOESM2_ESM.docx]
